# Additional Exergames to Regular Tennis Training Improves Cognitive-Motor Functions of Children but May Temporarily Affect Tennis Technique: A Single-Blind Randomized Controlled Trial

**DOI:** 10.3389/fpsyg.2021.611382

**Published:** 2021-03-15

**Authors:** Luka Šlosar, Eling D. de Bruin, Eduardo Bodnariuc Fontes, Matej Plevnik, Rado Pisot, Bostjan Simunic, Uros Marusic

**Affiliations:** ^1^Institute for Kinesiology Research, Science and Research Centre Koper, Koper, Slovenia; ^2^Institute of Human Movement Sciences and Sport, Department Health Sciences and Technology, ETH Zurich, Zurich, Switzerland; ^3^Division of Physiotherapy, Department of Neurobiology, Care Sciences and Society, Karolinska Institute, Stockholm, Sweden; ^4^Research Group in Physical Activity, Cognition and Behavior, Federal University of Rio Grande do Norte, Natal, Brazil; ^5^Department of Kinesiology, Faculty of Health Sciences, University of Primorska, Izola, Slovenia; ^6^Department of Health Sciences, Alma Mater Europaea – ECM, Maribor, Slovenia

**Keywords:** augmented and virtual reality, teaching/learning strategies, cognitive-motor learning, executive functions, tennis performance

## Abstract

This study evaluated the effects of an exergame program (TennisVirtua-4, Playstation Kinect) combined with traditional tennis training on autonomic regulation, tennis technique, gross motor skills, clinical reaction time, and cognitive inhibitory control in children. Sixty-three children were randomized into four groups (1st – two exergame and two regular trainings sessions/week, 2nd – one exergame and one regular training sessions/week, 3rd – two regular trainings sessions/week, and 4th – one regular training session/week) and compared at baseline, 6-month immediately post intervention and at 1-year follow-up post intervention. At 6-month post intervention the combined exergame and regular training sessions revealed: higher breathing frequency, heart rate (all *p*s ≤ 0.001) and lower skin conductance levels (*p* = 0.001) during exergaming; additional benefits in the *point of contact* and *kinetic chain* elements of the tennis *forehand* and *backhand* technique (all *p*s ≤ 0.001); negative impact on the *shot preparation* and the *follow-through* elements (all *p*s ≤ 0.017); higher ball skills (as part of the gross motor skills) (*p* < 0.001); higher percentages of clinical reaction time improvement (1st −9.7% vs 3rd group −7.4% and 2nd −6.6% vs 4th group −4.4%, all *p*s ≤ 0.003) and cognitive inhibitory control improvement in both congruent (1st −20.5% vs 3rd group −18.4% and 2nd −11.5% vs 4th group −9.6%, all *p*s ≤ 0.05) and incongruent (1st group −19.1% vs 3rd group −12.5% and 2nd group −11.4% vs 4th group −6.5%, all *p*s ≤ 0.001) trials. The 1-year follow-up test showed no differences in the tennis technique, clinical reaction time and cognitive inhibitory control improvement between groups with the same number of trainings per week. The findings support exergaming as an additional training tool, aimed to improve important cognitive-motor tennis skills by adding dynamics to the standardized training process. Caution should be placed to planning this training, e.g., in a mesocycle, since exergaming might decrease the improvement of specific tennis technique parts of the trainees. (ClinicalTrials.gov; ID: NCT03946436).

## Introduction

Early improvement in tennis technique has been important to optimize tennis success in professional players (The United States Tennis Association [USTA], 2009). A total of 70% of elite tennis players specialized early by selecting one sport while excluding others, at a mean age of 10.4 years with the aim to focus primarily on tennis technique enhancement (Jayanthi et al., 2011). However, those that engage in traditional training only in an early stage might encounter problems such as performing excessive repetitions that could lead to injuries (Dines et al., 2015), lack of motivation (Ochi and Kovacs, 2016), and a premature retirement of young professional players (Myer et al., 2015). Alternative training methods should, therefore, be further explored with the aim of improving tennis skills throughout attractive activities and without excessive physical stress in children.

Exergames, “technology-driven physical activities, such as video game play, that require participants to be physically active or exercise in order to play the game (Medicine, n.d.)” is widely used not only by children, but also among clinical populations (Benzing and Schmidt, 2017; Mat Rosly et al., 2017). Furthermore, exergame training supports motor skill competence in pre-school children (Gao et al., 2019) and is hypothesized having a positive effect on executive and visuo-spatial skills (Martin-Niedecken and Schättin, 2020). Other reported benefits are enjoyment, portability, challenge, psychological and social well-being, motivation, and different learning experiences (Joronen et al., 2016). The most recent meta-analysis revealed that despite the fact that exergames can contribute to a reduction in body mass index and body weight in non-clinical populations, they are unable to replace the physical activity of traditional sports (Gao et al., 2015; Oliveira et al., 2019). However, the benefits of exergames on fundamental and specific motor skills (Lubans et al., 2010; Barnett et al., 2012) during middle childhood (National Research Council, 1984) remains largely unclear with only three randomized clinical trials for children in middle childhood considered in a recent systematic review (Medeiros et al., 2017).

While there is no question about the importance of physical fitness attributes for tennis performance (Girard and Millet, 2009; Fernandez-Fernandez et al., 2014; Ulbricht et al., 2016) being successful in ball-sports also depends on cognitive skills (Vestberg et al., 2012). High and low league adult soccer players that are comparable based on their physical attributes, distinguish from each other in cognitive function with high league players presenting with better executive functions (EF) that relate to game performance compared to lower league players (Vestberg et al., 2012). The authors believe that these cognitive abilities help the players to make better decisions while applying their soccer technical skills. In tennis, the relation between technical skills and cognitive abilities (also defined as tactical skills) has been suggested one of the most significant factors that impact player’s performance (Kolman et al., 2019). In-game adaptations (visual search strategies), anticipatory and decision-making skills were found to be superior in advanced tennis players (Masters et al., 2008).

Tennis playing requires cognitive control for efficient decision making together with visual processing (visuo-spatial orientation) and specific tennis motor skills. Grigore et al. (2014) showed a positive correlation between the decision time and the sports performance (expressed through the ranking position) of elite tennis players aged between 15 and 17 years old. In addition, tennis players with enhanced decision-making skills, can use movement-pattern information to determine shot selection, reduce their response delay times and, hence, improve their stroke performance (Shim et al., 2005). In tennis, inhibitory control processes (identification of task-relevant information and the suppression of irrelevant stimuli) were shown to be superior compared to swimmers (Wang et al., 2013) and relevant to distinguish the level of athletes’ performance (Albuquerque et al., 2019). Interestingly, the interactive process can be simulated in exergames by combining motor and cognitive exercises in an attractive and progressive manner (McCaskey et al., 2018). Tennis exergame uses such exercise simulations that enables the player to stimulate attentional capacities, problem-solving and response speed directly embedded within the physical body movements to complete set tasks or actions, in response to visual cues (Sun, 2013). It is uncertain, however, whether this learning process transfers to real tennis performance and to what extent (positively or negatively) it affects the natural upgrade of motor stereotypes and competences (Magill, 2011); c.q. the overall performance. It seems important to monitor player’s psychophysiological responses when exergames are considered for long-term training purposes (Bronner et al., 2016; Cardona et al., 2016) to ensure the training principle of adequate progression is implemented.

The aim of the current study was to assess the effect of a 6-months exergame tennis intervention as an addition to regular tennis training on specific and generic tennis motor skills. First, the comparison of autonomic regulation responses during real and virtual environment playing at baseline, 6-month post intervention and 1-year follow-up post intervention was made. Additionally, we assessed how gross motor skills development, simple clinical reaction time (RT_clin_) and cognitive inhibitory control skills would be affected by the training interventions. We hypothesized that (i) the autonomic regulation responses would reveal higher emotional arousal and lower exercise intensity during exergame playing; (ii) the virtual intervention program would cause chronic adaptations to all parts of the tennis forehand and backhand technique; (iii) no difference on gross motor skills would be noted; and (iv) the combined tennis exergame and traditional playing groups would reveal greater intervention-related gains on simple clinical reaction time and cognitive inhibitory control skills compared to traditional tennis groups with an equal number of trainings per week.

## Materials and Methods

### Participants

Participants were recruited from the local tennis club (Tennis Club Koper, Slovenia, EU). Inclusion criteria were age between 7 and 9 years old, no more than 12 months of tennis experience (with regular participation in a training process). Players with injuries or long-term body impairments were excluded from the study. After predetermined exclusion criteria were applied, a detailed presentation of the study purposes and procedures to 67 participants and their parents were performed. We obtained 63 (36 boys, 27 girls, average age = 7.9 ± 0.9 years) signed written parental consents. The study was approved by the Republic of Slovenia National Medical Ethics Committee (no: 0120-631/2017/2) and registered on clinicaltrials.gov (NCT03946436).

**FIGURE 1:**
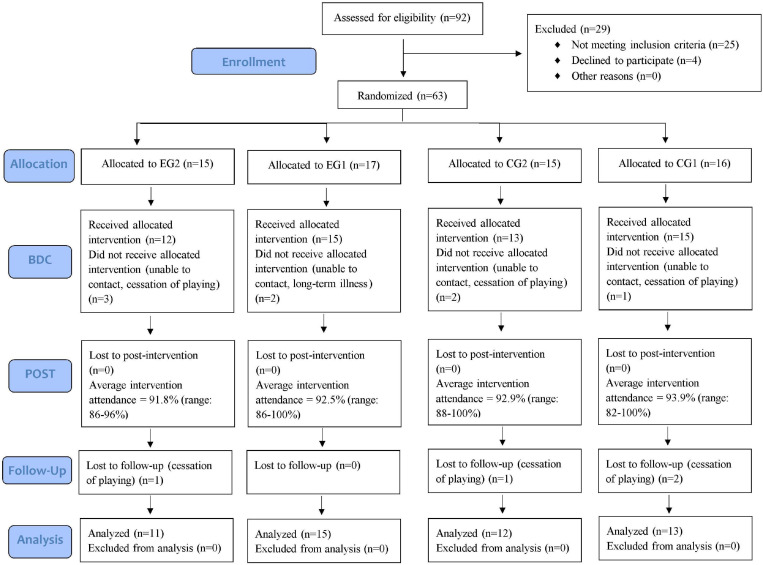
CONSORT flow diagram of study recruitment.

From the initial number of participants (*N* = 63), eight subjects voluntarily resigned from the study due to cessation of playing, or long-term illnesses prior to the baseline assessment (for details please see Figure 1). During the 1-year follow-up period following the post-intervention assessment, four additional players stopped playing tennis, hence they were not included in testing during follow-up.

### Research Design

Figure 1 presents the CONSORT flow chart of the study. Acknowledging a recommendation from International Tennis Federation (ITF) on the training intensity capacity of young novice players, 63 children were randomized into four groups using a single-blind design, (i) two exergames and two regular trainings/week (EG2), (ii) one exergame and one regular training/week (EG1), (iii) two regular trainings/week (CG2) and (iv) one regular training/week (CG1). The intervention period lasted for 6-months during which EG2 and EG1 groups were involved in a regular tennis training process, plus the additional intervention of tennis exergame. Both CG1 and CG2 groups followed solely the traditional tennis training program. Assessment was done at baseline (BDC), post-trainings (POST) and 1 year after the end of trainings sessions (FU). Additionally, to assess the participant’s exergame experience at BDC and FU, we used a self-designed questionnaire that was completed by children’s parents with their children.

### Intervention Activities

The tennis training conditions of the sessions were similar for all groups whereas participants had the same coach and played on the same court appropriate for children aged up to 9 years: red tennis court as suggested by the Lawn Tennis Association: 11 × 5.5 m court with, 0.8 m net height, low-compression felt ball, and 43–58 cm racquets; a portable tennis net and floor marking tape. Each practice session lasted for one hour, starting with a 10-min warming-up period, 40-min main activity (drills to improve hand-eye coordination, confidence with a racquet, and main groundstrokes techniques), and a 10-min cool down. The practice groups that had up to eight players were of mixed sex and not organized according to the study groups.

**FIGURE 2:**
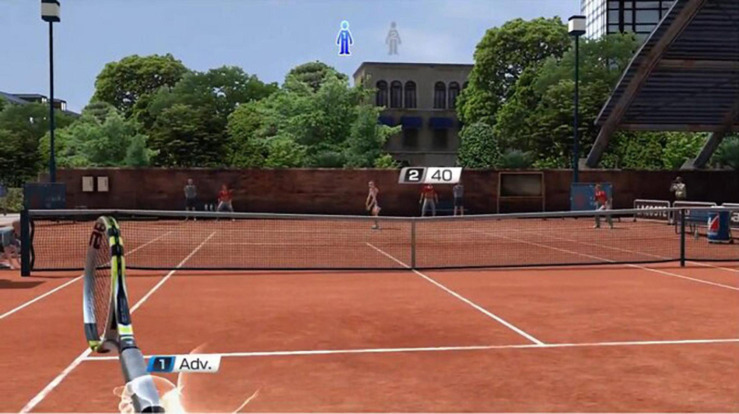
Exergame interface screenshot.

**FIGURE 3:**
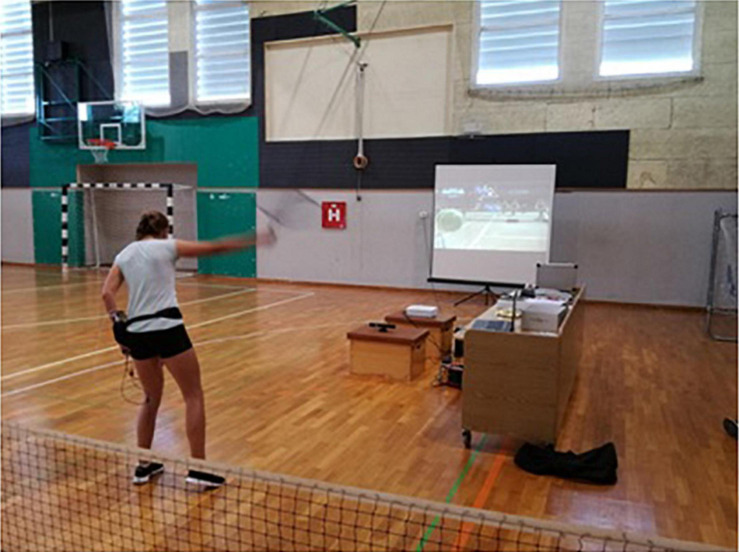
Exergame intervention.

The exergame intervention consisted of playing the active video game Virtua Tennis 4 (Sega Professional Tennis, Japan; Figure 2), on an Xbox 360 Kinect console (Microsoft, Washington). The Kinect motion sensing input device enables users to control and interact with the game without the need for a game controller. The combination of an RGB camera and a depth sensor provide full-body 3D motion capture (ranging limit 1.2–3.5 m), permitting to play through the console, by using body gestures and specific sport imitation movements. A projection screen (240 × 240 freestanding) was used to display the game through an image projector (NEC ME331X). Approximately 6 m^2^ of free ground surface space was provided to enable smooth movement (Figure 3). The distance between the player and the Kinect device was approximately 2 m, equal to the distance between the image projector and the screen. Two identical virtual playgrounds (as described above) were prepared to ensure intervention consistency.

The exergame intervention took place directly after the regular tennis practice sessions, either once (EG1) or twice a week (EG2). If tennis practice groups were mixed in a training session, no more than four participants at a time were involved in the exergame intervention. Each participant played on average 20 min per session. During the 6-months period a gradual increase in the complexity of tasks was provided following options offered by the exergame itself. During the first month, players practice shots with the ball machine, perfecting the groundstrokes timing from the baseline and the return. After that, they performed drills for hitting objects, improving attention and precision. The objects (mummies) were initially fixed (easy difficulty level) and then progressed to moving (medium difficulty level) around the court. When reaching the high difficulty level, mummies started to duplicate over time, with a different color for the duplicate. By hitting solely the red mummy you were able to eliminate all mummies with one shot. The last 3 months were used to perfect essential tennis skills playing matches against an avatar, against and together with (doubles) their peers. For each gaming option the difficulty level was progressed (easy, medium, and high) and the playing surface visual was changed (clay, grass, and hardcourt). Additionally, participants played the exergame using their tennis racquets to make the execution of the technique close to the real game.

The aim of each difficulty level was to achieve higher scores by using correct stroke technique. The game itself provides stroke feedbacks linked to swing velocity and timing. Added to this was the subjective assessment of whether the technique was positively evaluated by the tennis trainer during the intervention activities. When both criteria (point score and positive technique assessment) were met, the player was designated to play at the next level.

### Assessments

#### Autonomic Nervous System Responses

The assessment was performed according to a protocol by Knaepen et al. (2015), during random play in real vs virtual environment, using a NeXus-10 MKII physiological monitor (Mindmedia, Netherlands). The device consists of a breathing sensor (BF), worn directly on the participant’s chest that measures the number of breaths per minute; three surface electrodes were used to record heart rate (HR): one was placed two centimeters below the right clavicle between the first and second ribs, one was placed at the fifth intercostal space on the left mid-axillary line, and a ground electrode was placed on the right acromion. Skin temperature (ST) was measured using a thermistor point probe placed on the palmar surface of the middle phalanx of the left middle finger. Skin conductance (SC) was measured with two Ag–AgCl electrodes attached to the palmar surface of the middle phalanx of the left index and ring fingers with Velcro straps. In both conditions, players played with the ball machine for 5 min. To maintain the same intensity of playing, both conditions were performed at the same playing pace (approximately 25 balls/min). The conditions for the real game were prepared in the gym where the exergame intervention was performed (as described in the section “Intervention activities”).

#### Tennis Technique Rating Score for Children

The “Tennis rating score for children” (TRSC) was used to evaluate the backhand and forehand stroke technique execution. The TRSC represents a reliable (ICCr > 0.87 between raters and between days) and valid (convergent validity confirmed) measure when assessing technical skills in novice players. While performing shots players were recorded using a smartphone. Videos were later examined by an experienced tennis trainer using video processing programs (Adobe After Effects CC). The raters’ task was to evaluate the execution performed by players by comparing it with the description (with grading reference images) available for each stroke segment: shot preparation, acceleration, contact point, follow through and the kinetic chain (Šlosar et al., 2018). To better understand the impact that exergaming has on tennis technique, all segments were analyzed specifically for the backhand and the forehand stroke.

#### Test of Gross Motor Development

The test of gross motor development (TGMD-3) measures 13 fundamental motor skills, subdivided into two subscales: locomotor and ball skills. The two subscales scores are combined to form the Gross Motor composite. Performance evaluation was performed according to the Ulrich (2000) protocol. The assessment was performed only at BDC and POST. Only raw scores were taken into the analysis. All performances were videotaped and later analyzed by two independent researchers. The reliability of measurements was established according to Vernadakis et al. (2015). Specifically, 30% of children’s scores at BDC and POST were analyzed for between researchers (BR) and between days (BD; day1 and day10) reliability by using the intraclass correlations coefficient (ICC) (with 95% confidential intervals). ICC values were interpreted as proposed by Malcata et al. (2014) as follows: >0.99, extremely high; 0.99–0.90, very high; 0.75–0.90, high; 0.50–0.75, moderate; 0.20–0.50, low; and <0.20, very low. The ICC_(__2_,_1__)_ revealed a very high reliability between researchers (BR – day1) in both measurements (ICC_PRE–TEST_ = 0.894; ICC_POST–TEST_ = 0.877). Very high ICCs_(__3_,_1__)_ were also confirmed for between-day (BD – day1:day10) reliability (ICC_PRE–TEST_ = 0.881; ICC_POST–TEST_ = 0.891). In all ICCs, the 95% CI ranged from 0.863 to 0.899.

#### Simple Clinical Reaction Time Test (RT_clin_)

RT_clin_ was determined using a manual visuomotor task: the time needed to catch, by hand closure, a suspended vertical rigid measuring stick (Eckner et al., 2014). The participant sat with the dominant forearm resting on a desk and the hand positioned over its edge. The examiner released the 130 cm stick after a randomly determined time between 2 and 5 s after which the participant caught it as quickly as possible. After six practice trials, each participant performed eight acquisition data trials. The recorded distance on the stick was converted using the equation $t=\sqrt{2\,d\,/\,g}$ (*d* = catch point in cm; *g* = acceleration gravity), and reported as the average time (ms) needed to catch the stick after its release.

#### Cognitive Inhibitory Control

Cognitive inhibitory control was measured using a Simon task (Metzker and Dreisbach, 2011; Diamond, 2013). The assessment was performed on a laptop computer (ASUS N705UN-GC151, 17′′) and consists in identifying (as soon as possible), with the left or right button-press, whether a blue or red square shape appeared on a laptop screen. A total of 100 trials were presented, half congruent and half incongruent, with the order of trials randomized for each participant. When answering, the participants should not pay attention at the item location (left or right of fixation), however solely on its color. Due to the natural tendency to respond in the direction of task-relevant stimuli, reaction time is faster when the stimulus (red or blue square) is congruent with its location (left or right side of fixation). The amount of interference displayed by each participant was calculated as follows: mean reaction time on incongruent trials – mean reaction time on congruent trials. The difference score refers to as the “congruency effect”; where larger values are indicative of a less effective interference inhibition ability (Mullane et al., 2009). Additionally, answer accurateness (e.g., blue square – right keypress) in both congruent and incongruent trials was measured and reported.

### Statistical Analysis

All statistical tests were performed with SPSS 26.0 (IBM, Chicago, United States). All data are presented as mean and standard deviation. A one-way ANOVA was performed to check for any demographic differences between the groups. A Friedman test was used to analyze the differences in the (i) technique improvement between both experimental and control groups at BDC, POST, and FU. Two-way repeated measures ANOVA was conducted to evaluate the effect of interventions and measurements across time on the (ii) acute and chronic autonomic nervous system responses, (iii) gross motor development, (iv) RT_clin_ and (v) cognitive inhibitory control skills. The focus of the present analysis was on time, and possible group interactions. The level of significance was set at 0.05. The effect size for each analysis was assessed using the eta-squared statistic (partial η^2^) and interpreted as proposed by Cohen (1988): <0.01 = small, 0.01–0.06 = medium, >0.14 = large. A *post hoc* analysis with Wilcoxon signed-rank test was conducted with an applied Bonferroni correction, resulting in a significance level set at *p*<0.017. Furthermore, with a dependent samples *t*-test we compared the autonomic regulation between the virtual and the real environment at the BDC.

## Results

The anthropometric and demographic baseline characteristics of the final number of subjects are presented in Table 1.

**TABLE 1:** Participants baseline anthropometric data according to their groups.

	**EG2**	**EG1**	**CG2**	**CG1**	***P*-value**
*N* (Males/Females)	12 (8/4)	15 (11/4)	13 (9/4)	15 (6/9)	
Age (yr)	7.50.8	7.90.9	8.41	7.80.7	0.077
Body mass (kg)	27.24.6	31.611.1	35.96.3	30.95	0.055
Body height (cm)	131.58.7	135.110.8	1419.1	135.85.7	0.076
Body mass index (kg/m^2^)	15.82	16.83.2	17.92.1	16.61.6	0.183

*EG2 – group with 2 exergame and 2 regular tennis trainings/week; EG1 – group with 1 exergame and 1 regular tennis trainings/week; CG2 – group 2 regular tennis trainings/week; CG1 – group with 1 regular tennis training/week.*

The questionnaire about the children’s exergame experience at the BDC revealed almost equal percentages of occasional exergame players in all groups. The highest number of regular exergame players belonged to the EG1 and CG2. Most of the children had no experience with the tennis exergaming. At the FU four players (two in EG2 + one in EG1 + one in CG1) regularly played the tennis exergame. The attendance at the interventions were also recorded and ranged between 82 and 100% for all groups. Due to organizational problems, the attendance could not be calculated at FU.

### Acute and Chronic Autonomic Regulation

At the BDC all groups showed higher SC (level of arousal) while playing exergames compared to the real tennis game. A significant interaction effect for condition × time × group was noted in SC (*p* = 0.007, partial η^2^ = 0.201) and BF (*p* < 0.001, partial η^2^ = 0.208). At POST and FU, the HR and BF (indices of the effort level causing superior internal load) were higher in both experimental groups, as compared to the control groups (*p* < 0.001). The opposite occurred in SC, where the results showed higher values in both control groups as compared to the experimental groups (*p* = 0.001) (see Table 2).

**TABLE 2:** Autonomic regulation during real and simulated tennis practice after traditional and combined training interventions with exergame at baseline and post intervention in children.

		**BDC**				**POST**			**FU**					
	**Groups**	**Resting**	**Virtual Tennis**	**Real Tennis**	***p*-value**	**Resting**	**Virtual Tennis.**	**Real Tennis**	**Resting**	**Virtual env.**	**Real env.**	***P*time × group (η^2^)**	***P*condition × time (η^2^)**	***P*condition × time × group (η^2^)**
HR (beats per minute)	EG2	91.66	121.13.5	122.63.4	0.238	89.93.3	128.82.9	1293.5	89.43	127.64.1	1302.3	<0.001 (0.248)	<0.001 (0.442)	0.201
	EG1	91.84.7	123.74.8	1234.3	0.894	90.74.7	127.94.6	127.84.6	88.52.2	125.13.1	128.72.7			
	CG2	93.55.1	120.36.9	123.16.5	0.004*	913.5	121.75	129.15.2	90.32.4	1243.5	130.71.9			
	CG1	93.13.7	121.56	122.56.6	0.221	89.93.3	120.95.1	127.95.9	88.42.4	121.82.1	128.22.6			
p-value (groups)		0.622	0.661	0.926		0.671	<0.001*	0.709	0.219	0.001*	0.045			
SC (μS)	EG2	2.80.9	7.61.4	5.81.3	0.012*	2.50.5	5.61.2	6.51.1	2.50.6	5.11.1	6.11.2	0.007 (170)	<0.001 (0.215)	0.007 (138)
	EG1	2.81.1	7.31.1	5.51.1	< 0.001*	2.40.6	6.31.2	5.91.2	2.40.6	6.11.1	5.60.9			
	CG2	2.50.5	7.61.2	5.31.1	< 0.001*	2.40.7	7.21.1	5.90.9	2.30.5	6.41.3	5.90.8			
	CG1	3.21	6.81.3	5.61.2	0.003*	2.50.5	7.11	6.11.2	2.40.6	6.90.8	60.8			
*p*-value (groups)		0.202	0.323	0.846		0.895	0.001*	0.564	0.788	0.001*	0.490			
ST (°C)	EG2	25.71.4	282.2	29.31.7	0.092	25.13.2	30.92.5	29.61.8	24.81.1	31.11.8	30.31.7	0.437	<0.001 (0.216)	0.180
	EG1	251.3	28.41.2	29.41.5	0.025*	24.82.1	30.11.9	29.51.3	24.80.9	30.11.2	29.42.5			
	CG2	25.82.1	28.41.8	27.81.5	0.481	26.42.5	30.62.3	29.81.9	25.41.2	31.11.4	30.32.1			
	CG1	25.81.7	28.61.9	27.91.8	0.279	24.82.9	29.62.6	302.3	24.21.5	29.91.2	29.42.5			
*p*-value (groups)		0.384	0.784	0.035		0.480	0.599	0.904	0.108	0.071	0.594			
BF (breaths per minute)	EG2	22.52.9	26.81.7	26.41.9	0.643	21.31.2	32.51.8	32.21.9	22.41.1	31.21.6	33.31.3	<0.001 (0.346)	<0.001 (0.584)	<0.001 (0.208)
	EG1	21.72.6	25.91.8	26.31.2	0.497	21.41.7	31.11.9	31.11.8	21.31.9	29.71.4	32.11.9			
	CG2	20.62.2	26.21.8	26.11.3	0.980	21.11.6	27.21.7	32.22.1	20.81.7	27.51.8	33.41.8			
	CG1	21.82.7	25.52.1	26.82.2	0.017*	21.41.4	26.21.1	311.9	21.21.4	26.30.9	322.3			
*p*-value (groups)		0.414	0.594	0.410		0.806	<0.001*	0.236	0.100	<0.001*	0.138			

EG2 – group with 2 exergame and 2 regular tennis trainings/week; EG1 – group with 1 exergame and 1 regular tennis trainings/week; CG2 – group 2 regular tennis trainings/week; CG1 – group with 1 regular tennis training/week. BDC – baseline; POST – post intervention; FU – follow-up after 1 year; HR = heart rate; SC = skin conductance; ST = skin temperature; BF = breathing frequency. *p < 0.05.

### Tennis Technique (TRSC)

The Kruskal–Wallis test showed no difference between groups (*p*s > 0.05) at the BDC in all TRSC backhand and forehand evaluated elements. The forehand and backhand techniques showed an improvement over time in all four groups, Tables 3, 4 respectively. The *post hoc* analysis revealed that both experimental groups did not improve in elements related to the shot preparation and follow through phases (TRSC items: 1a, 4a, 4b, and 4c) at the POST in both strokes (*p* > 0.017). At the POST both control groups did not improve (*P* > 0.017) in specific elements related to the point of contact phase in the forehand and backhand stroke (TRSC items: 3a, 3c, 5a, and 5c). Only the CG1 did not improve (*p* > 0.017) in the kinetic chain phase (TRSC item: 5b-forehand; 5c-forehand/backhand) at FU.

**TABLE 3:** Forehand technique assessment with the “Tennis rating score for children” at baseline, post intervention and 1-year follow-up.

	**EG2**				**EG1**				**CG2**				**CG1**			
**TRSC item**	**BDC**	**POST**	**FU**	***p*-value**	**BDC**	**POST**	**FU**	***p*-value**	**BDC**	**POST**	**FU**	***p*-value**	**BDC**	**POST**	**FU**	***p*-value**
1a	2.1 ± 0.7	2.5 ± 0.5	3.9 ± 0.5*	<0.001	2.3 ± 0.5	2.5 ± 0.5	3.5 ± 0.5*	<0.001	2.7 ± 0.5	4.1 ± 0.7*	4.9 ± 0.3*	<0.001	2.1 ± 0.5	3.5 ± 0.5*	4.2 ± 0.4*	<0.001
1b	2.3 ± 0.8	3 ± 0.5*	4.2 ± 0.8*	<0.001	2.5 ± 0.8	2.9 ± 0.6*	3.8 ± 0.4*	<0.001	2.4 ± 0.9	3.7 ± 0.5*	4.3 ± 0.9*	<0.001	2.3 ± 0.8	2.9 ± 0.5*	3.8 ± 0.4*	<0.001
1c	2 ± 0.6	2.9 ± 0.7*	4.2 ± 0.8*	<0.001	2.1 ± 0.6	2.9 ± 0.4*	3.8 ± 0.4*	<0.001	2.3 ± 0.9	3.3 ± 0.9*	4.3 ± 0.9*	<0.001	1.9 ± 0.4	2.9 ± 0.3*	3.8 ± 0.4*	<0.001
2a	2.1 ± 0.7	2.9 ± 0.7*	4.2 ± 0.6*	<0.001	2.4 ± 0.5	2.6 ± 0.5	3.6 ± 0.4*	<0.001	2.3 ± 0.8	3.3 ± 0.7*	4.4 ± 0.6*	<0.001	2.1 ± 0.6	2.9 ± 0.5*	3.3 ± 0.4*	<0.001
2b	2.2 ± 0.8	3.7 ± 0.5*	4.7 ± 0.4*	<0.001	2.3 ± 0.5	3.1 ± 0.4*	3.4 ± 0.5	<0.001	2.1 ± 0.8	3.1 ± 0.7*	4.1 ± 0.6*	<0.001	2.1 ± 0.6	2.5 ± 0.5*	3.2 ± 0.7*	<0.001
2c	1.8 ± 0.4	2.8 ± 0.6*	4.3 ± 0.8*	<0.001	1.9 ± 0.4	2.7 ± 0.5*	3.4 ± 0.5*	<0.001	2 ± 0.7	2.9 ± 0.5*	4 ± 0.4*	<0.001	1.9 ± 0.4	2.6 ± 0.5*	3.3 ± 0.6*	<0.001
3a	2.3 ± 0.8	4.4 ± 0.5*	5 ± 0*0*	<0.001	2.4 ± 0.5	3.9 ± 0.7*	4.5 ± 0.5*	<0.001	2.5 ± 0.8	2.9 ± 0.5	3.8 ± 0.6*	<0.001	2 ± 0.6	2.4 ± 0.5	3.1 ± 0.3*	<0.001
3b	2.1 ± 0.7	2.8 ± 0.8*	3.9 ± 0.7*	<0.001	2.2 ± 0.7	2.9 ± 0.5*	3.8 ± 0.4*	<0.001	2.3 ± 0.8	2.9 ± 0.5*	4 ± 0.6*	<0.001	2 ± 0.6	2.5 ± 0.5*	3.4 ± 0.5*	<0.001
3c	2.5 ± 1.1	3.2 ± 0.6*	4.2 ± 0.5*	<0.001	2.7 ± 0.7	2.9 ± 0.6	3.7 ± 0.6*	<0.001	2.9 ± 1.1	3.5 ± 0.7	4.5 ± 0.6*	<0.001	2.3 ± 0.8	2.5 ± 0.5	3.5 ± 0.5*	<0.001
4a	2.8 ± 1	3.3 ± 0.6	4 ± 0.5*	0.001	2.7 ± 0.8	2.9 ± 0.7	3.3 ± 0.6*	0.001	2.8 ± 1	3.8 ± 0.6*	4.7 ± 0.4*	<0.001	2.5 ± 0.6	3 ± 0.6*	3.9 ± 0.6*	<0.001
4b	1.9 ± 0.8	2.4 ± 0.7*	3.4 ± 0.6*	<0.001	2.3 ± 0.6	2.5 ± 0.5	3.5 ± 0.5*	<0.001	2.3 ± 0.8	4 ± 0.6*	4.8 ± 0.5*	<0.001	1.8 ± 0.7	3.2 ± 0.7*	3.9 ± 0.6*	<0.001
4c	2.8 ± 0.4	2.9 ± 0.3	3.4 ± 0.5*	0.002	2.9 ± 0.5	3.1 ± 0.3	3.6 ± 0.5*	<0.001	2.8 ± 0.5	3.7 ± 0.7*	4.6 ± 0.5*	<0.001	2.7 ± 0.6	2.9 ± 0.4	3.8 ± 0.4*	<0.001
5a	2.3 ± 0.8	3.4 ± 0.5*	4.7 ± 0.5*	<0.001	2.5 ± 0.5	3.1 ± 0.4*	3.8 ± 0.6*	<0.001	2.5 ± 0.8	3.1 ± 0.7*	4.1 ± 0.5*	<0.001	2 ± 0.6	2.2 ± 0.4	3.1 ± 0.5*	<0.001
5b	2 ± 0.6	3.8 ± 0.4*	4.9 ± 0.3*	<0.001	2 ± 0.50.5	3.3 ± 0.4*	3.7 ± 0.4*	<0.001	2.2 ± 0.7	3.1 ± 0.7*	4 ± 0.6*	<0.001	1.9 ± 0.4	2.3 ± 0.5*	2.7 ± 0.6*	0.001
5c	1.8 ± 0.4	4 ± 0.4*	4.9 ± 0.3*	<0.001	2.1 ± 0.3	3.4 ± 0.5*	4.2 ± 0.6*	<0.001	2 ± 0.6	2.7 ± 0.5*	3.9 ± 0.5*	<0.001	1.9 ± 0.4	2.4 ± 0.5*	2.6 ± 0.5	0.001

EG2 – group with 2 exergame and 2 regular tennis trainings/week; EG1 – group with 1 exergame and 1 regular tennis trainings/week; CG2 – group 2 regular tennis trainings/week; CG1 – group with 1 regular tennis training/week. BDC – baseline; POST – post intervention; FU – follow-up after 1 year; 1 = shot preparation: 1a = arm position, 1b = backswing, 1c = stance; 2 = acceleration: 2a = weight transfer, 2b = hitting motion, 2c = racquet path; 3 = point of contact: 3a = height, 3b = body position, 3c = sweet spot; 4 = follow through: 4a = end position, 4b = racquet position, 4c = non-dominant arm position; 5 = kinetic chain: 5a = sequence of activated body segments, 5b = fluidity, 5c = timing. *Significant different from the previous measurement at *p* < 0.017.

**TABLE 4:** Backhand technique assessment with the “Tennis rating score for children” at baseline, post intervention and 1-year follow-up.

	**EG2**				**EG1**				**CG2**				**CG1**			
**TRSC item**	**BDC**	**POST**	**FU**	***p*-value**	**BDC**	**POST**	**FU**	***p*-value**	**BDC**	**POST**	**FU**	***p*-value**	**BDC**	**POST**	**FU**	***p*-value**
1a	2.2 ± 0.6	2.6 ± 0.5	4.2 ± 0.6*	<0.001	2.3 ± 0.5	2.5 ± 0.5	3.3 ± 0.5*	<0.001	2.4 ± 0.8	4.1 ± 0.7*	4.8 ± 0.4*	<0.001	2.1 ± 0.5	3 ± 0.6*	3.7 ± 0.5*	<0.001
1b	2.2 ± 0.8	2.9 ± 0.7*	4 ± 0.6*	<0.001	2 ± 0.5	2.7 ± 0.5*	3.5 ± 0.5*	<0.001	1.9 ± 0.8	3.1 ± 0.7*	4.2 ± 0.5*	<0.001	1.7 ± 0.5	2.7 ± 0.5*	3.5 ± 0.7*	<0.001
1c	1.5 ± 0.5	2.6 ± 0.5*	3.8 ± 0.6*	<0.001	1.5 ± 0.5	2.3 ± 0.5*	3.3 ± 0.4*	<0.001	1.8 ± 0.7	3.4 ± 0.8*	4.5 ± 0.8*	<0.001	1.5 ± 0.5	2.6 ± 0.5*	3.5 ± 0.4*	<0.001
2a	2 ± 0.6	2.8 ± 0.8*	4 ± 0.8*	<0.001	2.3 ± 0.5	2.9 ± 0.4*	3.5 ± 0.5*	<0.001	2.2 ± 0.8	3.3 ± 0.8*	4.3 ± 0.8*	<0.001	1.9 ± 0.7	2.7 ± 0.5*	3 ± 0.30.3	<0.001
2b	1.9 ± 0.5	3.7 ± 0.5*	4.7 ± 0.5*	<0.001	1.9 ± 0.4	3 ± 0.4*	3.5 ± 0.5*	<0.001	1.8 ± 0.6	3 ± 0.4*	3.9 ± 0.5*	<0.001	1.9 ± 0.4	2.3 ± 0.5*	3 ± 0.4*	<0.001
2c	2 ± 0.6	2.6 ± 0.7*	4.1 ± 0.7*	<0.001	2 ± 0.5	2.7 ± 0.5*	3.5 ± 0.5*	<0.001	1.8 ± 0.7	2.7 ± 0.5*	3.8 ± 0.4*	<0.001	1.9 ± 0.4	2.5 ± 0.5*	2.9 ± 0.6	<0.001
3a	2.4 ± 0.7	4.4 ± 0.5*	5 ± 0*	<0.001	2.4 ± 0.5	3.6 ± 0.5*	4.3 ± 0.4*	<0.001	2.4 ± 0.8	2.8 ± 0.4	3.8 ± 0.6*	<0.001	2 ± 0.6	2.3 ± 0.5	2.9 ± 0.4*	<0.001
3b	2 ± 0.6	2.8 ± 0.8*	3.9 ± 0.7*	<0.001	2.1 ± 0.6	2.9 ± 0.3*	3.8 ± 0.4*	<0.001	2 ± 0.9	2.9 ± 0.8*	3.9 ± 0.8*	<0.001	1.7 ± 0.5	2.3 ± 0.5*	3.1 ± 0.6*	<0.001
3c	2.3 ± 0.8	3.1 ± 0.5*	4.1 ± 0.5*	<0.001	2.3 ± 0.5	2.7 ± 0.5*	3.7 ± 0.4*	<0.001	2.5 ± 0.8	3.4 ± 0.5*	4.3 ± 0.5*	<0.001	2 ± 0.6	2.5 ± 0.5*	3.3 ± 0.5*	<0.001
4a	2.6 ± 0.8	2.8 ± 0.4	3.6 ± 0.5*	<0.001	2.5 ± 0.5	2.6 ± 0.5	3.4 ± 0.4*	<0.001	2.5 ± 0.8	3.7 ± 0.7*	4.7 ± 0.6*	<0.001	2 ± 0.6	2.9 ± 0.4*	3.7 ± 0.5*	<0.001
4b	1.7 ± 0.8	2.1 ± 0.5	3.2 ± 0.6*	<0.001	1.9 ± 0.8	2.3 ± 0.5*	3.3 ± 0.4*	<0.001	2.3 ± 0.8	3.8 ± 0.4*	4.8 ± 0.3*	<0.001	1.8 ± 0.4	2.9 ± 0.5*	3.7 ± 0.5*	<0.001
4c	2.6 ± 0.8	2.8 ± 0.4	3.5 ± 0.5*	0.001	2.8 ± 0.4	2.9 ± 0.5	3.2 ± 0.5	0.007	2.5 ± 0.8	3.7 ± 0.5*	4.6 ± 0.5*	<0.001	2.3 ± 0.7	2.8 ± 0.4*	3.6 ± 0.5*	<0.001
5a	2.2 ± 0.7	3.3 ± 0.5*	4.8 ± 0.4*	<0.001	2.3 ± 0.5	3.1 ± 0.3*	3.7 ± 0.5*	<0.001	2.3 ± 0.8	2.9 ± 0.3*	3.9 ± 0.3*	<0.001	1.9 ± 0.4	2.1 ± 0.3	3 ± 0*	<0.001
5b	1.8 ± 0.4	3.8 ± 0.4*	4.8 ± 0.4*	<0.001	1.9 ± 0.3	3.1 ± 0.5*	4 ± 0.5*	<0.001	1.8 ± 0.5	2.8 ± 0.4*	3.8 ± 0.4*	<0.001	1.9 ± 0.3	2.3 ± 0.5*	2.5 ± 0.5	0.001
5c	1.8 ± 0.4	3.9 ± 0.3*	4.9 ± 0.3*	<0.001	1.9 ± 0.4	3.3 ± 0.5*	4.2 ± 0.6*	<0.001	1.9 ± 0.5	2.7 ± 0.5*	3.6 ± 0.5*	<0.001	1.8 ± 0.4	2.1 ± 0.4	2.5 ± 0.5	0.001

EG2 – group with 2 exergame and 2 regular tennis trainings/week; EG1 – group with 1 exergame and 1 regular tennis trainings/week; CG2 – group 2 regular tennis trainings/week; CG1 – group with 1 regular tennis training/week; BDC – baseline; POST – post intervention; FU – Follow-up after 1 year; 1 = shot preparation: 1a = arm position, 1b = backswing, 1c = stance; 2 = acceleration: 2a = weight transfer, 2b = hitting motion, 2c = racquet path; 3 = point of contact: 3a = height, 3b = body position, 3c = sweet spot; 4 = follow through: 4a = end position, 4b = racquet position, 4c = non-dominant arm position; 5 = kinetic chain: 5a = sequence of activated body segments, 5b = fluidity, 5c = timing. *Significant different from the previous measurement at *p* < 0.017.

### Gross Motor Development (TGMD-3)

At the BDC no differences were found in TGMD-3 mean score (*p* = 0.234) between the groups. At POST we found main effect for time (*p* < 0.001, partial η^2^ = 0.985) and time × group (*p* = 0.021, partial η^2^ = 0.172). An additional analysis (Table 5) revealed no interaction in the locomotor skills (*p* = 0.685), and a main effect of time × group for the ball skills (*p* < 0.001, partial η^2^ = 0.293).

**TABLE 5:** Overall, ball and locomotor skills TGMD-3 interaction analysis.

	**Overall skills**					**Ball skills**					**Locomotor skills**				
	**BDC**	**POST**	***P*time (η^2^)**	***P*group (η^2^)**	***P*time × group (η^2^)**	**BDC**	**POST**	***P*time (η^2^)**	***P*group (η^2^)**	***P*time × group (η^2^)**	**BDC**	**POST**	***P*time (η^2^)**	***P*group (η^2^)**	***P*time × group (η^2^)**
			<0.001 (0.985)	0.306	0.021 (0.172)			<0.001 (0.990)	0.175	<0.001 (0.293)			<0.001 (0.961)	0.415	0.685
EG2	63.1 ± 5.1	97.5 ± 0.9				28.7 ± 3.1	52.9 ± 0.7				34.4 ± 2.3	44.6 ± 0.7			
EG1	66.4 ± 5.1	96 ± 1.7				30.8 ± 2.6	51.1 ± 1.2				35.6 ± 2.5	44.9 ± 0.8			
CG2	66.5 ± 3.7	97.7 ± 1.4				31.3 ± 2.4	52.8 ± 0.7				35.1 ± 1.8	44.9 ± 0.9^#^			
CG1	65.1 ± 4.1	95.5 ± 1.8				30.3 ± 2.5	51.1 ± 1.3				34.7 ± 2.1	44.4 ± 0.9			
*p*-value (groups)	0.234	0.001				0.112	< 0.001*				0.531	0.332			

EG2 – group with 2 exergame and 2 regular tennis trainings/week; EG1 – group with 1 exergame and 1 regular tennis trainings/week; CG2 – group 2 regular tennis trainings/week; CG1 – group with 1 regular tennis training/week; BDC, baseline; POST, post intervention. *p < 0.001.

Examining the TGMD-3 total score, showed that the EG2 had a higher percentage of improvement (54.7%) over the intervention period. Other groups had similar improvements: 46.9 in CG1, 46.7% in CG2, and 44.6% in EG1.

### Simple Clinical Reaction Time Test (RT_clin_)

**FIGURE 4:**
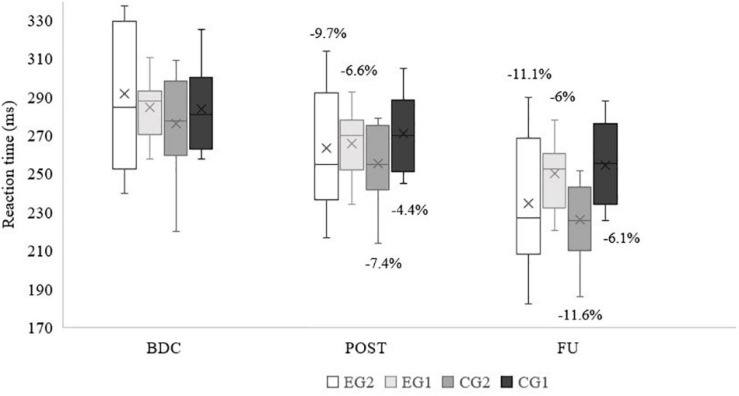
Clinical reaction time across time (percentages of improvement, averages, medians, first and third quartiles, minimum, and maximum values are shown). EG2 – group with 2 exergame and 2 regular tennis trainings/week; EG1 – group with 1 exergame and 1 regular tennis trainings/week; CG2 – group 2 regular tennis trainings/week; CG1 – group with 1 regular tennis training/week; BDC – baseline; POST – post intervention; FU – Follow-up after 1 year.

No difference between the groups were found in the BDC RT_clin_ measurements (*p* = 0.535). Both a significant main effect for time (*p* < 0.001, partial η^2^ = 0.970) and time × group interaction (*p* < 0.001, partial η^2^ = 0.700) were noted. At POST and FU both experimental groups had a higher percentage of improvement as compared to the control groups with the identical number of trainings per week (Figure 4).

### Cognitive Inhibitory Control

**FIGURE 5:**
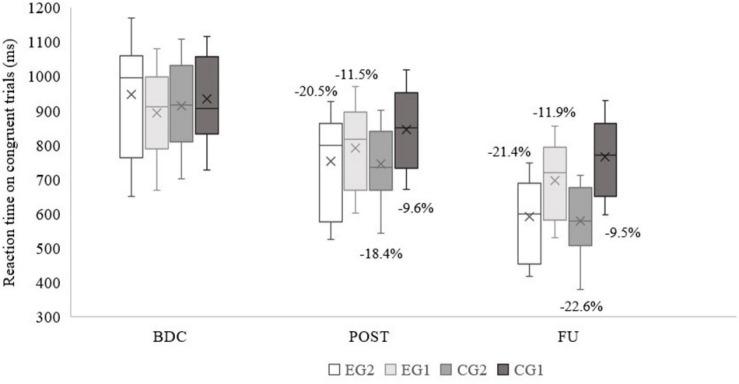
Simon task measurements on congruent trials (percentages of improvement, averages, medians, first and third quartiles, minimum, and maximum values are shown).EG2 – group with 2 exergame and 2 regular tennis trainings/week; EG1 – group with 1 exergame and 1 regular tennis trainings/week; CG2 – group 2 regular tennis trainings/week; CG1 – group with 1 regular tennis training/week; BDC – baseline; POST – post intervention; FU – Follow-up after 1 year.

**FIGURE 6:**
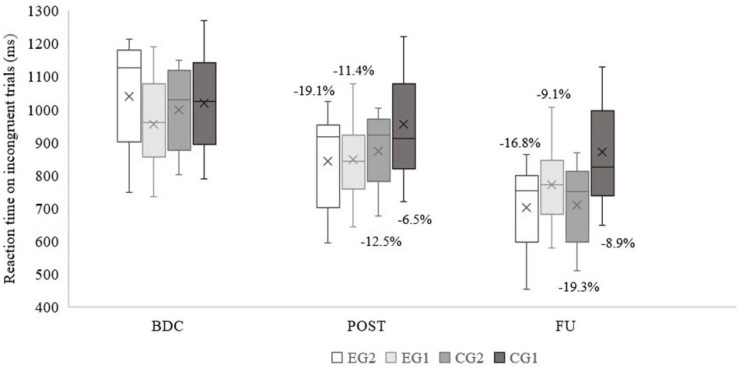
Simon task measurements on incongruent trials (percentages of improvement, averages, medians, first and third quartiles, minimum, and maximum values are shown). EG2 – group with 2 exergame and 2 regular tennis trainings/week; EG1 – group with 1 exergame and 1 regular tennis trainings/week; CG2 – group 2 regular tennis trainings/week; CG1 – group with 1 regular tennis training/week; BDC – baseline; POST – post intervention; FU – Follow-up after 1 year.

There were no group differences at the BDC in reaction time of Simon tasks at the congruent trials: *p* = 0.770; and incongruent trials: *p* = 0.506. Main effects of time and time × group were found in both congruent (*p* < 0.001, partial η^2^ = 0.974 and *p* < 0.001, partial η^2^ = 0.780, respectively) and incongruent trials (*p* < 0.001, partial η^2^ = 0.974 and *p* < 0.001, partial η^2^ = 0.803, respectively). In Figures 5, 6 the mean reaction time per group and the percentage of improvement are shown. EG2 had the highest percentage of improvement at POST in both trials. Both experimental groups exhibit almost the same improvement across trials (post-intervention). The control groups, specifically CG1, performed worse at the incongruent trials, which resulted in lowest percentage of improvement.

The response accuracy across the trials showed no main effect of time × group (congruent trials: *p* = 0.137; incongruent trials: *p* = 0.186). The “congruency effect” (Table 6) revealed a main effect of time (*p* < 0.001, partial η^2^ = 0.331), and time × group (*p* < 0.001, partial η^2^ = 0.292). Both control groups significantly enlarge the gap at the POST. The same was observed also for both experimental groups at the FU.

**TABLE 6:** “Congruency effect” results.

	**BDC**	**POST**	**FU**	***P*time (η^2^)**	***P*group (η^2^)**	***P*time × group (η^2^)**
				<0.001 (0.331)	0.080	<0.001 (0.292)
EG2	93.764.3	90.562.8	110.261.1^#^			
EG1	63.342.7	57.147	74.847.1^#^			
CG2	84.944.5	127.944.6^#^	129.442.8			
CG1	85.249.3	109.960^#^	105.356.9			
*p*-value (groups)	0.440	0.009*	0.065			

EG2 – group with 2 exergame and 2 regular tennis trainings/week; EG1 – group with 1 exergame and 1 regular tennis trainings/week; CG2 – group 2 regular tennis trainings/week; CG1 – group with 1 regular tennis training/week; BDC – baseline; POST – post intervention; FU – Follow-up after 1 year. *p < 0.05. ^#^Significantly different from previous measurement at *p* < 0.05.

## Discussion

The aim of this study was to determine whether specific exergame playing with different session frequencies in addition to traditional tennis training would affect general tennis techniques, gross motor skills, visual processing, and cognitive inhibitory control skills in children. First we assessed the autonomic regulation responses at the baseline, post 6-month intervention and at 1-year follow-up post intervention during virtual and real tennis game to determine whether the additional exergames were able and reach an appropriate level of exergame difficulty (Cardona et al., 2016). When exergames are used, either as a complement to conventional training or in isolation, it is important to ensure monitoring of players reaction following long-term involvement with the training (Bronner et al., 2016). Based on the favorable measures observed in the EG groups when compared to the CG groups it seems fair to assume we were able and maintain trainee’s attention through adequate progression of difficulty levels with the exergame. The low attrition rate in our training sample seems to further underpin this observation. The used inclusion/exclusion criteria precluded meaningful disparity between participants in relation to complexity progression and intensity of playing.

A combined exergame and traditional tennis intervention negatively affects the shot preparation and the follow through phase of the tennis technique as observed in both evaluated strokes. An additional development was visible in the point of contact and the kinetic chain. Exergames further stimulate the development of gross motor skills that demonstrate efficient throwing, striking, and catching movements. Gender differences were negligible with relation to skills tests and practice results. Visual processing and the cognitive inhibitory control skills revealed higher percentages of improvement after the combined training interventions. At the 1-year follow-up no differences between groups with similar number of trainings per week were found.

### Acute and Chronic Autonomic Nervous System Responses

To the best of our knowledge, our study is the first to examine the long-term effects of a combined exergame and traditional tennis playing on autonomic nervous system responses. In accordance with Pusenjak et al. (2015), the NeXus-10 MKII physiological monitor was used to measure autonomic nervous system responses to a longitudinal training intervention. In addition to long-term adaptations, we obtained children’s responses data during a single bout of exercise. As hypothesized, throughout the acute exposure, all groups revealed higher emotional arousal when playing the exergame. Arousal and excitement have been shown to be predictors of cognitive engagement (Boucsein, 2012), suggesting a possible contribution of the virtual game as a complementary learning strategy. It can be speculated that the combination of physical and cognitive load needed to play the exergame explains, at least in part, the effect on HR (Young and Benton, 2015). The hypothesis of lower exercise intensity during exergame playing has been refuted, as results revealed higher values in HR and BF as an effect of chronic adaptation to exergame playing. These results could be linked to the experience, permitting to play on a higher effort level causing a higher internal load. A recent meta-analysis (Gao et al., 2015) found similar results reporting higher HR during the exergame playing compared to stationary biking. The same results were reported comparing 30 min of brisk walking on a treadmill with a Nintendo Wii Fit intervention (Douris et al., 2012). In a review article Biddiss and Irwin (2010) reported the possibility to perform low- to moderate-intensity exercise training (50–70% HRmax), while playing specific exergames. The results are in line with ours, as the HR in both experimental groups at the POST range between 61 and 68% HRmax. The maximum HR was set at 200 beats per minute in all subjects (Bar-Or, 1983). At POST and FU the SC was lower for combined exergame and regular tennis intervention groups, suggesting a more comfortable feeling with the virtual environment which allowed them to push themselves harder during the exercise. Previous research has provided evidence that an acute bout of exergame could provide the recommended intensity of cardiorespiratory fitness. However, none of them performed a direct comparison between performing the same sport in different environments, e.g., in real life setting and in a virtual environment (McNarry and Mackintosh, 2016; Viana et al., 2018).

### Tennis Technique Improvement

We evaluated the forehand and backhand technique performance using the TRSC (Šlosar et al., 2018), and we excluded the serve stroke from the present assessment, as its performance is too demanding for children in middle childhood and is, therefore, only partially addressed and performed at this age.

If we consider the improvement in the TRSC made by the control groups, as the natural technique development followed by a regular tennis training process in young children, then our findings confirm the hypothesis that an additional longitudinal exposure to exergames, causes chronic adaptations related to almost all parts of the tennis technique in both evaluated strokes (backhand and forehand). Negatively affected are the shot preparation and the follow through phase, which both developed mainly through the analytical course of learning. Both experimental groups seem to hurry in the preparation for the next shot, paying less attention to proper stance and arm positions. In the exergame the level of control the user executed over the actions of the avatar during the game, is deducted from more prominent results in simple reaction time and timing of the hand swing. If the hand does not swing at the right time, the avatar would fail to shoot the ball. However, in the current model, the avatar’s positions are not taken into consideration for a successful stroke. Players adaptations on the current model reflected in less or even no improvement in the shot preparation and follow through phase, and a faster progress in the point of contact and kinetic chain phase. According to Crespo et al. (2004) an improvement in active attention, focus, projection, and reception skills are fundamental to perform the elements, point of contact and kinetic chain, on a higher level. Therefore, the exergames seems to emphasize the game-based learning approach, which affirm the importance of learning through practice, in match-like drills and actual play, instead of practicing strokes with the same repetitive technical execution.

Testing during follow-up showed the transient effect of exergames and their impact during the intervention, since the average improvements in technique of both experimental and control groups (considering those with the respective number of trainings per week), are almost equal in the point of contact, follow through, and kinetic chain phases. The absence of studies with a separate testing during the follow-up, hinder comparison, and the potential link-effect with the participant’s young age.

According to Pill (2017) the dynamics of the tennis game is not associated with the common practice sessions that minimize variations in speed, spin, bounce of the ball and placements on the court. The same problem also affects novice players (Reid et al., 2013). Thus, exergames may have the potential to be implemented in a tennis training process to further develop specific game-related skills that are often under-addressed during practice with novice players.

### Test of Gross Motor Development (TGMD-3)

The TGMD-3 assessment was performed only at BDC and POST. Our hypothesis of no differences between the groups for gross motor skills is only partially confirmed, as a time × group interaction was found in the ball skills subtest (e.g., throwing, catching, kicking, two/one hand striking, and dribbling) but not in the locomotor skills. Our results can be just partially compared with those of Vernadakis et al. (2015) in which the same main effect was noted in the object control skills (previously titled ball skills in the TGMD-2), after an 8-week exergaming vs a traditional training intervention and a no-training group. The exergames (in our case tennis virtual game) involved constant adjustments based on distance, target points and the opponent. Continuous learning seems to be positively affected to the general skills, like timing and attention, which result in higher ball skills. Based on our and related findings (e.g., Vernadakis et al., 2015), we can assume that incorporating the exergames into children’s real sport activities and training processes, would help them to achieve recommended levels of fundamental movement skills.

### Simple Clinical Reaction Time (RT_clin_)

The results seem to support our hypothesis indicating that a superior training effect in groups combining forms of training can be expected by confirming the highest percentage of improvement seen in EG2 at the POST time-point. The EG1 performed the same as the CG2. At FU groups with the same number of trainings per week exhibited almost similar improvements. These results suggest the presence of a positive exergame contribution to the shortening of the reaction time at POST. The improvement possibly derived from the exergame playing model as already explained in section “Tennis Technique Improvement.”

The outcome of the simple reaction time might vary according to the technique of assessment (digital or analog). In the scientific literature we were not able to find studies assessing simple reaction time in children using the analog reaction time stick. Our BDC results are however comparable with those of Wilkinson and Allison (1989) in which the simple reaction time in children under the age of 10 was digitally evaluated. However, there were no studies to evaluate reaction time after prolonged tennis or exergame training. Thus, further research is required.

### Cognitive Inhibitory Control

While playing exergames, an interaction is established between the user and the virtual environment. The interaction involves the processing of multiple sensory information (visual, auditory, and somatosensory) that generate a sensory flow. This can occur through task stimuli with predictable items (closed tasks) or random item tasks (opens tasks). The first requires simple capabilities as they do not require the perception of the changing environment. With open tasks, the constantly changing environment requires greater attention, ability to inhibit disturbing elements, decision making, and more accurate reaction time. The required EFs are of great importance to correctly perform the movements resulting from open tasks stimuli. Individuals who regularly perform open-ended tasks exhibit improved EF and faster reaction time (Taddei et al., 2012). Most of the currently available exergames can be regarded as open-ended tasks that have an ability to improve certain cognitive skills required in ball games (Vestberg et al., 2012, 2017). Hence, cognitive inhibitory control skills are one of the most positively affected sensory abilities (Monteiro-Junior et al., 2016). Our results support the hypothesis of advantages for the combined training approach. A time × group interaction was present in all evaluated reaction time parameters. As seen in the RT_clin_, higher percentages of improvement were observed in both experimental groups compared to control groups with the same number of trainings per week. This occurred in both congruent and incongruent trials at the POST. At FU it could be concluded that the groups (comparing those with the respectively number of trainings per week) maintained the same percentage of improvement, suggesting the effectiveness of the 6-month exergaming to a reaction time. To our knowledge this is the first study that evaluates effects of prolonged exergame playing on cognitive inhibitory control skills in children. Our results can be partially compared with those of Flynn and Richert (2018) and Best (2012), who both demonstrate the effect of an acute bout of exergame on visual perceptual skills using a Flanker test (better accuracy and reaction time). In a similar study design, O’Leary et al. (2011) found no exergame benefits on cognitive control. Benzing et al. (2016) and Staiano et al. (2012) assessed the EF skills including visual-perceptual skills using a paper-and-pencil measure called the Delis-Kaplan Executive Function System. In both, the exergame intervention outperformed a no-play group and simple aerobic activity. In Morton and Harper (2007), a study that used the Simon task to investigate a bilingual advantage in middle childhood, the mean response times in congruent and incongruent trials are in line with those of our participants at the BDC, where the results ranged between 900 and 1,000 ms. Furthermore, these results seem to confirm findings of improved EF in older adult exergame trainees (Eggenberger et al., 2016; Schättin et al., 2016).

Interestingly, the “congruency effect” showed larger values in both control groups at the POST. These results are due to a higher improvement in the congruent over the incongruent trials during the intervention period. These circumstances may be explained by the analytical teaching approach with novices, as mentioned before. As already explained, common practice sessions wrongly minimize in ball speed, bounce, spin, and placements on court variations (Reid et al., 2013; Pill, 2017). Compared to some other sports like soccer or basketball, the tennis needs more practice sessions (stroke repetitions) to perform real game matches. Repetitive actions (linked to practice sessions with novice) seem to be less effective on the development of cognitive inhibitory control skills compared to exergames. At FU we confirmed larger “congruency effects” in both experimental groups. At this time, the control groups maintain the same gap, stopping the negative trend observed during the intervention period.

### Strengths, Limitations, and Future Directions

To our knowledge, the present study is the first assessing chronic effects of exergame alongside training in real conditions. Moreover, it is the first study to use FU tests to better understand and confirm long-term effects of exergame intervention.

We did not perform the state-of-the-art neurophysiological measurements in the current study; hence we are unable to determine which processes were optimized by the exergame intervention. Reuter et al. (2019) found that cognitive processing speed (P3 latency) is the only significant predictor of response speed in children at the Flanker test. The authors further note that cognitive control (frontal N2 and P3 amplitude), and cognitive update (parietal P3) are important predictors of response accuracy. According to Reuter et al. (2019) findings, exergame may had primarily improved information processing speed (P3 latency). As there were no differences between the groups in the accuracy response, we cannot assert to have equally affected the cognitive control (frontal N2 and P3 amplitude) and cognitive updating (parietal P3). Further studies with neurophysiological measures (e.g., EEG) as well as expanded battery of neuropsychological measurements should therefore support our behavioral findings. Despite the already assessed cognitive inhibitory skills (Simon task), subsequent studies should expand the investigation on working memory and EF to broaden and substantiate the already acquired knowledge. This study is a response to the general interest of coaches, parents and the general public in the effects of playing exergames alongside tennis training, as well as a general lack of scientific data regarding virtual reality games and the development of EF and complex movement.

## Conclusion

The diverse stimuli derived by the virtual reality game seem to positively affect various skills such as timing, interference/conflict processing, and reaction time, hence the overall game performance of tennis. The development in game dynamics requires alternative activities that can add variety to training sessions. Tennis trainers could try to vary their training process by using the exergame as a complementary training tool with novice players to achieve additional training effects related to executive functions. Attention must be paid to the quality of practice under virtual conditions, especially during the first tennis development phase (6–8 years) when no technique recognition patterns are already established. Advices during shot preparation and follow-through phases could possibly prevent the negative exergame effects on tennis technique. Involving novice players in an in-game context may control their attitudes that predetermine their interpretations and increase subsequent confidence in game events.

## Data Availability Statement

The original contributions presented in the study are included in the article/supplementary material, further inquiries can be directed to the corresponding author/s.

## Ethics Statement

The studies involving human participants were reviewed and approved by the Republic of Slovenia National Medical Ethics Committee (no: 0120-631/2017/2). Written informed consent to participate in this study was provided by the participants’ legal guardian/next of kin.

## Author Contributions

LŠ, RP, BS, and UM designed the research. LŠ and UM performed the experiments and drafted the manuscript. LŠ, BS, and UM analyzed the results of the experiments. LŠ, EB, EF, MP, RP, BS, and UM edited and revised the manuscript. LŠ, EB, EF, MP, RP, BS, and UM approved the final version of the manuscript. All authors contributed to the article and approved the submitted version.

## Conflict of Interest

The authors declare that the research was conducted in the absence of any commercial or financial relationships that could be construed as a potential conflict of interest.
